# Hydrodynamic Mixing Tunes the Stiffness of Proteoglycan‐Mimicking Physical Hydrogels

**DOI:** 10.1002/adhm.202001998

**Published:** 2021-05-04

**Authors:** James P. Warren, Danielle E. Miles, Nikil Kapur, Ruth K. Wilcox, Paul A. Beales

**Affiliations:** ^1^ School of Chemistry University of Leeds Leeds LS2 9JT UK; ^2^ School of Mechanical Engineering University of Leeds Leeds LS2 9JT UK; ^3^ Institute of Medical and Biological Engineering University of Leeds Leeds LS2 9JT UK; ^4^ Astbury Centre for Structural Biology University of Leeds Leeds LS2 9JT UK; ^5^ Bragg Centre for Materials Research University of Leeds Leeds LS2 9JT UK

**Keywords:** biomimetic materials, biopolymer networks, gel mechanics, network connectivity, self‐assembling peptides, shear rheology

## Abstract

Self‐assembling hydrogels are promising materials for regenerative medicine and tissue engineering. However, designing hydrogels that replicate the 3–4 order of magnitude variation in soft tissue mechanics remains a major challenge. Here hybrid hydrogels are investigated formed from short self‐assembling *β*‐fibril peptides, and the glycosaminoglycan chondroitin sulfate (CS), chosen to replicate physical aspects of proteoglycans, specifically natural aggrecan, which provides structural mechanics to soft tissues. Varying the peptide:CS compositional ratio (1:2, 1:10, or 1:20) can tune the mechanics of the gel by one to two orders of magnitude. In addition, it is demonstrated that at any fixed composition, the gel shear modulus can be tuned over approximately two orders of magnitude through varying the initial vortex mixing time. This tuneability arises due to changes in the mesoscale structure of the gel network (fibril width, length, and connectivity), giving rise to both shear‐thickening and shear‐thinning behavior. The resulting hydrogels range in shear elastic moduli from 0.14 to 220 kPa, mimicking the mechanical variability in a range of soft tissues. The high degree of discrete tuneability of composition and mechanics in these hydrogels makes them particularly promising for matching the chemical and mechanical requirements of different applications in tissue engineering and regenerative medicine.

## Graphical abstract

Selective control of the physicochemical properties of hydrogel biomaterials allows more precise tuning for the requirements of the native biology. Here it is demonstrated that the vortex mixing time during the self‐assembly of hybrid hydrogels formed from chondroitin sulfate and self‐assembling peptides influences their mesoscale structure, giving broad tuneability of mechanical properties within a range commensurate with natural soft tissues.

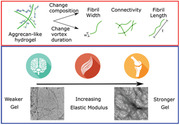

## 1 Introduction

**Figure 1:**
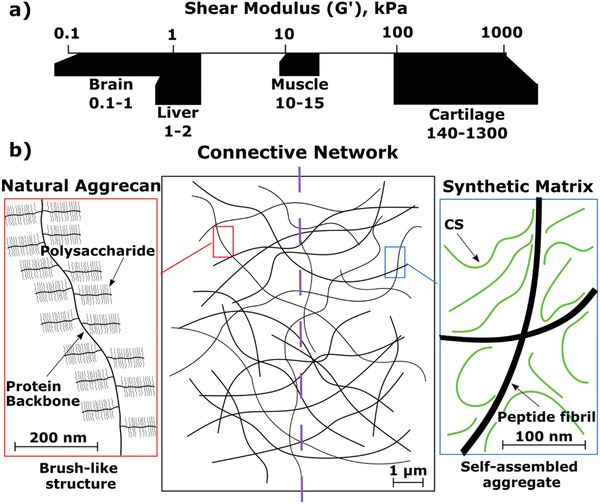
a) Soft tissue mechanics can range over many orders of magnitude: the shear modulus ranges of several soft tissue types are represented.^[^ ^2^, ^3^ ^]^ b) Schematic representation of the similarities and differences between the natural and synthetic structures of aggrecan and the self‐assembled peptide (SAP) hydrogels. Natural aggrecan adopts brush‐like structures on the micron scale. The synthetic SAP matrix is a marco‐molecular composite of interacting peptide fibrils and chondroitin sulfate (CS) polymers (≈50 kDa). Both structures form an interconnected matrix, the aggrecan molecule within the extracellular matrix of native tissue, while the SAP matrix is a fibrillar hydrogel.

The soft tissues in the human body exhibit a wide range of mechanical properties from ≈1 kPa in very soft materials such as brain to over 1 MPa in load‐bearing tissues such as cartilage (**Figure** **1**a).^[^ ^1^, ^2^, ^3^ ^]^

These mechanical properties are often intrinsic to function across length scales from molecular level organization and dynamics up to the biomechanics of the whole organ. It is well established that both biochemical cues and the mechanical environment are fundamental to cell behavior and functionality.^[^ ^4^, ^5^ ^]^ Therefore, in the development of biomaterials for implants or regenerative therapies, control over both the chemical composition and mechanical properties is of huge importance to the performance of the therapy.

Load‐bearing soft tissues such as articular cartilage, meniscus and intervertebral disc have presented challenges in mimicking using manufactured scaffolds due to the need to replicate the mechanisms for supporting load through both the solid and fluid phases while maintaining a biochemically favorable environment. These tissues contain macromolecules that are critical to their biomechanical performance. One of the most abundant is aggrecan, a proteoglycan aggregate comprising a protein backbone that provides structural rigidity and attached polysaccharide molecules that project out into a “brush‐like” structure.^[^ ^6^, ^7^ ^]^ These polysaccharides are known as glycosaminoglycans (GAGs), of which one of the most abundant is chondroitin sulfate (CS).^[^ ^8^, ^9^ ^]^ They are poly‐anionic due to a variety of sulfation patterns and play a pivotal role in maintaining the osmotic swelling pressure within the tissue, which is necessary to provide the fluid load support.

With aging, disease or trauma, there can be a loss of the proteoglycan structures, which in turn affects the mechanical performance of the tissue.^[^ ^10^, ^11^ ^]^ One potential therapeutic route to the restoration of healthy function is to augment the tissue with a biomaterial that can mimic the roles of these natural biopolymers. This approach has led to the design and development of natural and synthetic macromolecular constructs that are either polymeric or “polymer‐like” in their behavior.

A particularly promising class of materials are self‐assembling peptides (SAPs) which form a hydrogel matrix within which GAGs can be either entrapped or bound to mimic the overall structure and function of aggrecan. Self‐assembling peptides are a class of nanomaterials that are able to undergo spontaneous self‐assembly through thermodynamically and kinetically driven processes to form fibrillar networks. Once these networks reach a critical concentration, a self‐supporting hydrogel forms. Due to the different parameters that can drive self‐assembly, a plethora of SAPs have been developed, based on the formation of one of two different secondary structures: *α*‐helices based coiled‐coils , ^[^ ^12^, ^13^, ^14^, ^15^ ^]^ and *β*‐sheet forming peptide classes such as Fmoc dipeptides,^[^ ^16^, ^17^, ^18^, ^19^ ^]^ RADA, ^[^ ^20^, ^21^ ^]^ MAX,^[^ ^22^, ^23^, ^24^, ^25^ ^]^ and P_11_‐X peptides,^[^ ^26^ ^]^ or self‐assembly of peptide amphiphiles.^[^ ^27^, ^28^ ^]^ The intrinsic and tuneable properties of SAPs along with their ability to be functionalized with a variety of motifs or markers have enabled them to be utilized within the field of regenerative medicine, either as carrier scaffolds for various cell types or as structural biomaterials.^[^ ^29^, ^30^ ^]^

Each of these different SAPs can be tuned through altering external stimuli such as pH, temperature and ionic strength of the solvent in which the SAPs are dissolved. Through altering these stimuli, critical energetics and mechanisms of self‐assembly can be controlled. These changes can cause differing fibrillar morphologies, altered gelation times and different material properties.^[^ ^31^, ^32^ ^]^ The properties can also be further enhanced through combination with GAGs and other biopolymers such as collagen to give rise to even “stronger” hydrogels.^[^ ^33^, ^34^ ^]^ The interactions between the GAGs and SAPs are not fully understood but indications and theory point to effects such as electrostatics, entanglement, and charge screening.^[^ ^35^, ^36^, ^37^, ^38^, ^39^ ^]^

Here we focus on the P_11_‐X *β*‐strand forming peptides. This class of SAPs is based on an eleven amino‐acid sequence with alternating hydrophobic and hydrophilic side chains surrounding an aromatic central region that undergo hierarchical, nucleated self‐assembly.^[^ ^26^, ^40^ ^]^

When in the presence of GAGs, P_11_‐X peptides self‐assemble into hydrogels which have been proposed to mimic some physical aspects of proteoglycans, specifically aggrecan, through the formation of a supramolecular structures composed of P_11_‐X peptide fibrils decorated by GAG chains (Figure 1b).^[^ ^34^, ^41^, ^42^ ^]^ GAGs promote the self‐assembly of these gels through a combination of electrostatic and hydrogen bonding interactions with the peptide, lowering the critical aggregation concentration.^[^ ^34^ ^]^ The peptides still form *β*‐fibrils, while the GAGs are proposed to decorate along these peptide backbones.

The mechanical properties of SAP‐GAG hydrogels can be controlled through the composition and abundance of the individual components, for example the amino acid composition of the peptide and the mixing ratio of peptide:GAG.^[^ ^34^ ^]^ This attribute means the hydrogels have potential to be tuned to mimic the mechanical properties of a large range of soft tissues. However, gels are nonequilibrium states of matter and so their structure, and therefore their mechanical properties, are also strongly dependent upon the sample history.^[^ ^24^, ^43^ ^]^

The prospect of using hydrogel processing parameters in their preparation to independently tune the chemical composition and mechanical properties would be highly desirable in engineering optimal biomaterials for specific applications. Vortex fluid mixing devices have previously been used to control material properties, for example by the exfoliation of graphene from graphite.^[^ ^44^, ^45^ ^]^ Here we show that the duration of fluid shear forces in the initial mixing of the peptides and CS can result in a two order of magnitude variation in the shear modulus of the resultant gel, at fixed composition. This tuneability alongside variation of the peptide:GAG ratio in a P_11_‐12/CS hydrogel allows a range of gel shear moduli to be attained that are representative of the broad range of soft tissue mechanics found in the human body.

## 2 Results

A range of peptide‐GAG hybrid hydrogels are formed and oscillatory shear rheology is used as a measure of the mechanical properties of the gel. Gels are further characterized by fourier transform infrared spectroscopy (FTIR) to provide information on the molecular‐scale structure of these physical hydrogels and transmission electron microscopy (TEM) to observe structure on the mesoscale. The latter measurements aim to allow correlation of a gel's mechanics to its cross‐length scale structure.

### 2.1 Vortex Time Modulates Gel Rheology at Fixed Composition

We find that the stiffness of gels is strongly dependent upon the vortex mixing time used in the gel formation protocol. Therefore, we systematically investigate this phenomenon for five different vortex mixing times (30, 60, 120, 300, 600 s) at three different gel compositions (P_11_‐12:CS of 1:2, 1:10, 1:20).

**Figure 2:**
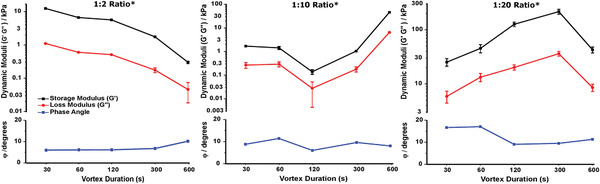
Rheological measurements of the storage and loss moduli and the phase angle of the different P_11_‐12: CS ratios at different vortex durations. (Data represented as mean, *n* = 3 per point, two‐way ANOVA tests with Tukey post‐hoc analysis were performed, *p* ≤ 0.05, * indicates a significant difference in dynamic moduli between two or more vortex durations at a given composition, error bars = S.D)

The effect of varying the duration of vortexing on the stiffness of the different gels is assessed by carrying out a frequency sweep at a constant strain on a rheometer (**Figure** **2**). Across all the compositions and durations of vortexing, these gels have a phase angle ranging between 5° and 18°, demonstrating the elastic nature of these materials. At the lowest mixing ratio (1:2), increasing the vortex duration decreases the stiffness of the hydrogels formed: the measured storage modulus (*G′*) of the hydrogels decreases by nearly two orders of magnitude—from 1.2 × 10^4^ to 3 × 10^2^ Pa. At the maximum duration, the hydrogel loses all cohesion and is better described as a viscous liquid than a self‐supporting gel.

For our intermediate composition ratio (1:10), we observe a more complex trend in gel mechanics with increasing vortex time. Self‐supporting gels formed for all vortex times. Initially, a similar shear‐thinning effect as seen for the 1:2 composition ratio is observed: the shear elastic modulus decreases by approximately one order of magnitude over a fourfold increase in vortex time from 30 to 120 s (1.7 × 10^3^ to 1.4 × 10^2^ Pa). This is followed by a shear‐thickening regime as the vortex time further increases by fivefold to 600 s, the shear elastic modulus of the hydrogel rises sharply by two orders of magnitude (to 4.6 × 10^4^ Pa). These latter gels were the stiffest across the range of vortex times investigated at this composition.

Further increasing the CS content to a composition ratio of 1:20 resulted in much stiffer hydrogels than observed at the lower mixing ratios. However, we still find that the gel mechanics are significantly tuneable by the duration of vortex mixing. As the vortex mixing time is increased from 30 to 300 s the hydrogel shear thickens with the shear elastic modulus increasing by an order of magnitude from 2.5 × 10^4^ to 2.2 × 10^5^ Pa. However further increasing the vortex time to 600 s results in a sharp, order of magnitude drop in gel stiffness to 4.3 × 10^4^ Pa. The trend for the viscous modulus follows that of the elastic modulus for all gel compositions studied.

These studies reveal a complex history‐dependence of the resultant mechanical properties in the preparation of these hydrogels. While increasing the CS content, on average, increased the shear elastic modulus of the gels, the mechanical properties at fixed composition varied significantly across orders of magnitude with different vortex times. This variation resulted in some crossover of mechanical properties of the hydrogels at specific vortex durations, particularly apparent when comparing 1:2 and 1:10 compositions. The 1:2 peptide:CS hydrogel is stiffer than the 1:10 composition for vortex durations of 120 s and below, whereas the 1:10 peptide:CS composition is stiffer for vortex durations of 300 s or above. Shear thickening and shear thinning behavior is seen depending on the hydrogel composition and a transition between these two regimes can occur for a particular composition above a critical vortex time.

This suggests an intricate role for the timescale of hydrodynamic shear from vortex mixing in controlling the assembly and structure of the gel fibrils and their network, the details of which determine the final mechanical properties of the hydrogel. These effects could influence the molecular scale interactions between the peptides and GAGs in the assembly of individual fibrils and/or the mesoscale network structure in terms of the length or diameter of individual fibrils and their interconnectivity in self‐supporting networks. We investigate these aspects further by FTIR (molecular interactions) and TEM (mesoscale structure).

### 2.2 Vortex Time Does Not Affect Molecular‐Scale Self‐Assembly

The core structure of the fibrils in these hybrid peptide‐GAG hydrogels is formed by the peptide, which self‐assembles into anti‐parallel *β*‐sheet structures. Under appropriate conditions, the peptides alone can self‐assemble into self‐supporting gels. However the GAGs facilitate self‐assembly by speeding up gelation times and lowering the critical concentration for self‐assembly.^[^ ^34^ ^]^ We use FTIR to probe the molecular‐scale interactions between peptides in the hydrogel by measuring the changes in the amide ^I^ IR region (1800–1600 cm^−1^). This region relates to the peptide backbone and is sensitive to subtle changes in the environment. Two regions of the IR spectrum correspond to antiparallel *β*‐sheets, the ranges 1613–1630 and 1682–1690 cm^−1^. The integrated area under the curve (AUC) of each peak in these two regions allowed the *β*‐sheet content to be calculated as a percentage of the total AUC in the amide ^I^ IR region for each sample. Representative examples of the resulting processed spectra are shown in Figure S2a–f in the Supporting Information.

**Figure 3:**
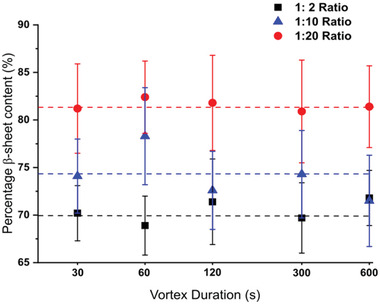
FTIR analysis of the *β*‐sheet content of each P_11_‐12: CS ratio at the different vortex durations. The content was measured through the amide I absorption area and the percentage attributed to anti‐parallel *β*‐sheet formation. (Data represented as mean percentage content, *n* = 3 per point, error bars = experimental S.D)

While analysis of the *β*‐sheet content of our hydrogels revealed a dependence on composition through an increase in CS content causing an increase of up to ≈10% in the *β*‐sheet content, no dependence was seen with varying vortex time at a fixed composition (**Figure** **3**). The average *β*‐sheet content of 1:2 hydrogels was 70.4 ± 3.4%, increasing to 74.2 ± 4.5% at 1:10 composition and 81.5 ± 4.6% at 1:20 composition. Our values for the 1:10 composition are consistent with previously published data of approximately 75%.^[^ ^34^ ^]^ Increasing CS composition leading to an increased *β*‐sheet content is also consistent with previously reported work,^[^ ^41^ ^]^ where the increase in CS promotes rapid self‐assembly and gelation. We find that CS promotes more rapid gelation and increases the formation of anti‐parallel *β*‐sheet structures, resulting in stiffer gels.

The lack of significant differences in *β*‐sheet content between the different vortex durations at fixed composition indicates that the changes measured in gel mechanical moduli are not due to changes in the molecular‐scale self‐assembly of the peptide‐GAG constituents that form the hydrogel. By elimination, this suggests that the underlying explanation for the observed history‐dependent mechanics lies within the mesoscale structuring of the gels, which we investigate by TEM.

### 2.3 Mesoscale Structure Correlates with Gel Mechanics with Two Distinct Mechanistic Regimes

**Figure 4:**
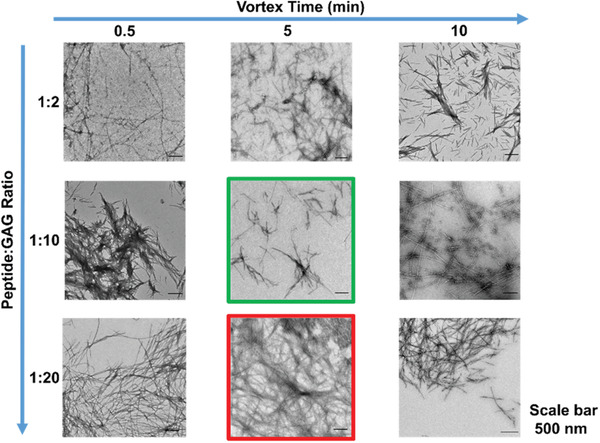
Negatively stained TE images (Magnification: 4000×) of each P_11_‐12: CS ratio at three different vortex durations. The green and red boxes indicate images of the weakest and strongest gels formed in this group respectively.

Negative‐stain TEM reveals differences in the mesoscale interfibrillar networks of the hydrogels (**Figure** **4**).(Additional images and higher magnification images are shown in Figure S3 in the Supporting Information.) The 1:2 P_11_‐12:CS gels exhibit thin fibrils (<30 nm in width) with a comparatively low number of apparent junction points forming between fibrils. As the vortex duration increases, these fibrils become shorter (<500 nm), bundled into tape/ribbon‐like structures with the apparent interconnectivity between the different bundles decreasing to a point where connections become very limited. This decrease in connectivity correlates with the decreasing shear elastic modulus of this gel composition with increasing vortex time. The hydrodynamic shear forces imparted during vortex mixing reduce the connectivity of the fibrillar network by breaking up the thin filaments into shorter segments that cannot form as many junctions, weakening the bulk mechanical strength. The breakdown of these fibrils at this composition suggests that the individual filaments are mechanically less stable, possibly due to weaker intermolecular interactions in the filament at this low CS concentration.

Increasing the CS content to 1:10 P_11_‐12: CS, the fibrillar network is more interconnected with longer individual filaments (>500 nm). Increasing the duration of vortex mixing does not cause dramatic changes in the fibril length, suggesting stronger intramolecular interactions at the higher CS content prevent significant break up. This leads to only small changes in the apparent interconnectivity of the network. These small changes result in the decreasing storage moduli, which in Figure 4 is highlighted with the green box as the weakest gel in the figure. The exception occurs after 10 min of vortexing, where the apparent fibrillar width and interconnectivity increases significantly, consistent with the sharp increase in stiffness of these gels. This longer mixing time may cause the untwisting of the fibrils, resulting in less rigid filaments which in turn may facilitate movement of the individual filaments within the forming network, allowing a greater number of collisions between fibrils and thereby forming a greater number of junction points.

At the highest CS composition (1:20 P_11_‐12:CS), longer (≥1 µm) and thicker (≥40 nm) fibrils form. However, the most notable difference across this gel composition is the increasing connectivity of the network with increasing vortex time, again correlating with increasing shear elastic modulus of the gels. The increase in gel stiffness reaches a critical point above 5 min vortexing, which is highlighted by the red box in Figure 4 as having the highest storage moduli compared to the other systems in this figure. A significant drop in gel shear elasticity is seen at 10 min vortex mixing, which correlates with a decrease in overall connectivity in the gel network. If longer vortex times allow greater collisions and hence junctions between filaments in the forming network, a critical threshold could be reached if high local connectivity in some regions of the gel resulted in regions of high density fibrils which are then connected by regions of lower connectivity due to depletion of the available fibrils in that region of the network. This could lead to an overall reduction in the bulk mechanical strength of the gel.

**Figure 5:**
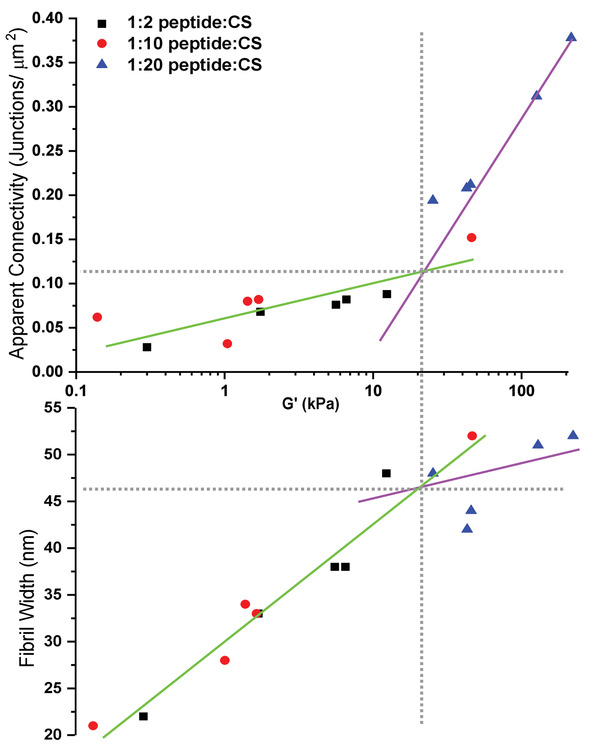
The relationship between the storage modulus of each hydrogel with the relative connectivity of the fibrillar network and the fibril width (nm). The horizontal grey dashed line indicates the structural threshold for the change in mechanism. The vertical grey dashed line indicates the value of the storage modulus at which the transition between mechanisms occurs.

Quantitative analysis of fibril width and connectivity reveals that the elastic moduli of these gels, across the three compositions studied, fall onto a master curve with two distinct mechanistic regimes (**Figure** **5**). At low *G*′, increasing elasticity of the network is dominated by the increase in fibril width, while at high *G*′, the fibril width begins to plateau and increases in the elastic modulus are dominated by a more pronounced increase in the apparent connectivity of the fibrillar network. The cross‐over between these two regimes occurs at ≈20 kPa.

## 3 Discussion

Previous work on P_11_‐12:CS hybrid hydrogels have reported that an increase in CS content within the P_11_‐12 matrix promotes the formation of stiffer gels through formation of thicker, longer fibrils.^[^ ^34^, ^41^ ^]^ As well as increasing the rate of gelation, CS has been shown to lower the critical concentration (c*) for self‐assembly of P_11_‐12 peptides.^[^ ^34^ ^]^

**Figure 6:**
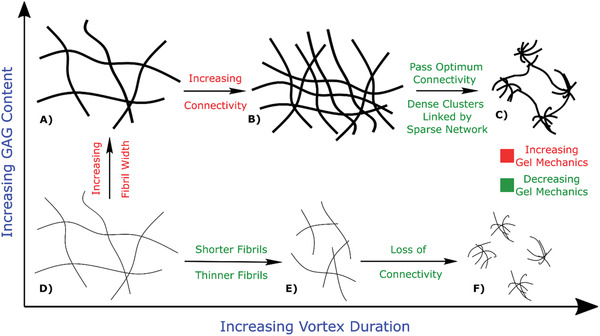
Schematic summarizing the dominant effects on gel structure (and hence its mechanics) from variation of GAG content (CS) and vortex mixing time. Text in green highlights structural effects that increase gel mechanics; text in red highlights structural effects that decrease gel mechanics. At low GAG content, increased vortexing reduces the length and width of fibrils, weakening the gel up to a point where percolating connectivity in the gel network becomes lost. At high GAG content, thicker fibrils are more stable to break‐up by vortexing due to strong intrafibril cohesive interactions. Instead, increased vortex mixing allows fibrils to rearrange into networks or greater connectivity and hence greater stiffness. However, this connectivity can exceed an optimum threshold where dense clusters become linked by more sparse networks that then weaken the overall gel mechanics.

Gel mechanics are a complex interplay between the properties of their fundamental building blocks and the networks that they form,^[^ ^46^, ^47^ ^]^ where an understanding is required of how experimental variables control these parameters.^[^ ^48^ ^]^ Overall, the complex trends in gel mechanics that we observe with increasing CS composition and vortex time can be explained by the competition of three effects of increasing the mixing time (**Figure** **6**): (i) increasing fibril width, (ii) break‐up of individual fibrils due to hydrodynamic stresses, and (iii) increasing collisions and hence connections between fibrils.^[^ ^49^ ^]^ Increasing CS content appears to increase the cohesive strength of individual fibrils under the hydrodynamic shear forces of vortex mixing and promote thicker fibrils to form, reducing their propensity to break down into shorter segments. At low CS content, fibril break‐up dominates, weakening the gels.^[^ ^50^ ^]^ As the individual fibrils become thicker and more stable at higher CS content, the network‐enhancing effect of greater collisions during longer mixing starts to become more dominant, enhancing the connectivity and hence strength of the gel.^[^ ^51^ ^]^ However, this can reach and surpass an optimal network connectivity, where further increasing the mixing time leads to local high density regions in the network. These local regions contain fibrils that have become shorter, potentially through fragmentation, and aggregated to form higher density regions. These regions of shorter fibrils are connected by lower density regions of the network, depleted in fibrils, that weaken the overall bulk mechanics of the gel.^[^ ^49^ ^]^ We find that the gel stiffness increases in two distinct regimes, where increasing fibril width is the dominant mechanism at shear moduli below ≈20 kPa and enhanced connectivity of the hydrogel network dominates in the high shear moduli regime.

The peptide‐GAG hydrogels investigated in this work are thought to be a reasonable minimal imitation of the proteoglycan filaments of extracellular matrices that provide the mechanical structure in soft tissues.^[^ ^52^, ^53^, ^54^ ^]^ By controlling the duration of hydrodynamic shear forces during the gelation process, we can vary the stiffness of these gels by up to two orders of magnitude at fixed composition. Therefore, composition and mechanics can be individually selected over a broad range of accessible shear moduli. Notably, across the three compositions studied, a range of approximately three orders of magnitude in shear elastic moduli are attained in a range that matches that found in natural soft tissues as shown in Figure 1. In brain tissue, the GAG content is very low, <1 µg mg^−1^ of dry tissue. At this low concentration of GAGs and the low shear elastic modulus, a composition of 1:2 or even lower would be ideal to closely match these two parameters.^[^ ^55^ ^]^ In comparison, cartilage tissue has a very high GAG content, >200 µg mg^−1^ of dry tissue. This high concentration of GAGs and high shear elastic modulus would require a composition of 1:20 or greater to replicate these two parameters.^[^ ^56^, ^57^ ^]^ Mechanobiology is now well established as a major contributor to the development and functionality of cells and tissues.^[^ ^3^, ^58^, ^59^ ^]^ While in this work we used an acellular system to investigate the fundamental relationship between composition and vortex duration during preparation, the inclusion of cells is possible in these systems. The complex interaction between composition and vortex duration highlights the need for individual optimization in this and other systems to identify the ideal conditions for each sample composition. The inclusion of cells would introduce additional parameters which would require further optimization of the system to the desired mechanics. Therefore peptide‐GAG hydrogels are a promising biomaterial for wide‐ranging applications in regenerative medicine and tissue engineering.

## 4 Experimental Section

### Sample Preparation

Lyophilized peptide (P_11_‐12, Ac‐SSRFOWOFESS‐NH_2_) was weighed out (20 mg, 0.014 mol, purchased from CS Bio) into glass vials and reconstituted in 130 mM unbuffered NaCl solution (pH 7.4) in triplicate (for FTIR samples, D_2_O was used as the base solvent rather than H_2_O) (500 µL). Lyophilized CS (*M*_W_ ≈ 50 kDa, ratio of CS‐4:CS‐6 1:3, purchased from ScanDroitin) was weighed out (12, 60, or 120 mg) into glass vials and reconstituted in 130 mM NaCl solution in triplicate (for FTIR samples, D_2_O was used as base solvent rather than H_2_O) (500 µL). The two components—peptide and CS were added to a single vial in the order of peptide then CS using a Gilson pipette. Different ratios of peptide: CS were made up using the different amounts of CS weighed out. A 1:2 ratio was obtained using 20 mg P_11_‐12 and 12 mg CS. The ratio was based around molar ratios of the peptide molecule to the molecular weight of the CS dimer. A 1:10 ratio was obtained using 20 mg P_11_‐12 and 60 mg CS and 1:20 was obtained using 20 mg P_11_‐12 and 120 mg CS. The samples were then vortexed for varying times (30, 60, 120, 300, and 600 s) at 2500 rpm on a vortex mixer (Stuart SA8). A homogenous, nematic gel forms, which was then stored in a dark place, to avoid UV damage or photo bleaching of peptide, at room temperature for 24 h. Samples were then tested within 2–3 h after storage. In order to ensure sample consistency between different experiments, the TEM grids were prepared on the same day that the rheological measurements are performed on each sample.

### Rheological Measurements

All the rheological measurements were performed on a Malvern Kinexus Pro rheometer with a plate‐plate geometry of diameter 25 mm and gap height of 0.25 mm with 250 µL sample required per test. Temperature was maintained at 37 °C and a solvent trap was used to minimize evaporation of the peptide samples. Loaded samples were left for 15 min to equilibrate before testing.

To measure the dynamic moduli of each hydrogel, the linear viscoelastic region (LVER) was first identified through amplitude sweeps over the shear strain range of 0.01% to 100% at frequencies of 1 and 20 Hz (See Figure S1a–c in the Supporting Information for example plots at a given composition and duration). At these frequencies, a shear strain of 0.1% was found to consistently lie within the LVER where the elastic modulus (*G*′) and viscous modulus (*G*′′) remain constant. These settings were away from the sensitivity limits of the instrument where noise was observed in the measurements. No drops in the elastic modulus or peaks in the viscous modulus were observed indicating the absence of sample slip.

The dynamic moduli of the hydrogels were then measured as a function of frequency between 1 and 20 Hz using the identified strain of 0.1%.

### FTIR Spectroscopic Analysis

Samples were placed between CaF_2_ crystals and their spectra acquired with a Thermo Scientific Nicolet 6700 FTIR spectrometer. Spectra were averages of 32 scans recorded at room temperature whilst purging with dry air. Blank solvent spectra were subtracted from the sample trace, the baseline corrected and the spectra smoothed. Processed spectra were band fitted using the Peak Resolve routine in OMNIC 7.3 SP1 (Thermo Electron Corporation, Loughborough, UK). The FTIR spectrum was fitted using Gaussian functions to measure the amount of random coil and other organized secondary structures such as *β*‐sheets and *β*‐turns or hairpins. The *β*‐sheet signal was detected at 1615 cm^−1^ with a weak peak measured 1681–1686 cm^−1^ which indicates the *β*‐sheet structure is arranged in an antiparallel structure as previously reported in Miles et al (Miles et al. 2016). In every spectrum, there is a peak around 1640 cm^−1^ which correlates to the presence of monomeric peptide which coexists with the self‐assembled *β*‐sheet structure—mainly random coil.

### Transmission Electron Microscopy

Transmission electron microscopy was carried out using a JEOL 1400 electron microscope. Electron microscope (EM) grids (copper hexagonal 400 mesh) precoated with a carbon film from mica sheets were prepared in‐house. Peptide gels were used at the following concentrations: P_11_‐12 = 14 × 10^−3^ m, CS = 14 × 10^−3^ m for 1:2 ratio, 70 × 10^−3^ m for 1:10 ratio and 140 × 10^−3^ m for 1:20 ratio. The peptide gels remained in contact with the grids for one minute, the excess buffer solution was then removed by wicking on filter paper. The grids were negatively stained by absorption of 2 % w/w aqueous uranyl acetate solution for 20 s. The excess was removed, again via wicking with filter paper, and left to air dry. Images were obtained within 24 h to avoid artefacts and destruction of the sample with the TEM operating at 80 kV accelerating voltage.

### Dimensional Analysis of Images

The width, length, and connectivity of the fibrillar network of each gel were analyzed using ImageJ software to obtain a quantitative measure of the fibrillar widths, lengths, and number of junction points. Each TEM image was sectioned into 500 × 500 nm grids and the number of junctions between connecting fibrils was measured, then the average for each image taken. The grid was overlaid using a plug‐in for ImageJ which allows the area of each grid to be 500 × 500 nm Another plug‐in, developed externally by (DiameterJ^[^ ^60^ ^]^), allows the number of junction points within the fibrillar network to be counted. The average number of junctions per grid area was recorded, where wrongly identified junctions were eliminated if they meet specific criteria

- The junction point is in fact aggregation of salt from the buffer solution
- The junction point is parallel running fibrils
- The junction point is parallel running fibrils, where the resolution is unable to allow decision as to whether contact is made or not

**Figure 7:**
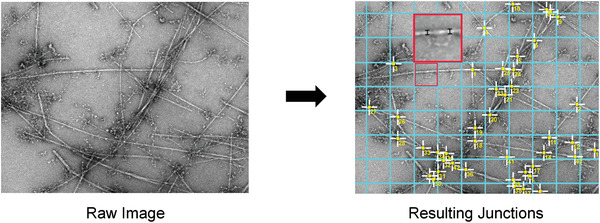
Images illustrating the process carried out in ImageJ to analyze the dimensions and connectivity within each hydrogel's fibrillar network. a) A raw negatively stained micrograph. b) Using the Grid analysis plugin, a 500 × 500 nm grid is overlaid. Using an external plugin, the junction points of the fibrillar network are identified by ImageJ. c) Highlighted in the enlarged area (red), two examples of measuring the fibril width are shown.

Five images were taken for each specimen and the average of each were then further averaged to obtain a global average of the sample. An example is illustrated in **Figure** **7**.

### Statistical Analysis

Fifteen different hydrogel systems were used resulting from three different compositions (1:2, 1:10, and 1:20), with five different vortex durations. Each hydrogel system was produced in triplicate (*n* = 3), where each sample was tested independently. Data were reported as mean ± standard deviation. Two‐way ANOVA tests with Tukey post‐hoc analysis were performed (*p* ≤ 0.05). Statistical analysis was carried out using Origin 2019 software (OriginLab Corporation, USA).

## Conflict of Interest

The authors declare no conflict of interest.

## Supporting information

Supporting Information

## Data Availability

The data that support the findings of this study are openly available in The White Rose Repository at http://doi.org/10.5518/975.
