# Supplementary material for: Hydrodynamic Mixing Tunes the Stiffness of Proteoglycan‐Mimicking Physical Hydrogels
Source: Adv Healthc Mater. 2021 May 4;10(11):2001998. doi: 10.1002/adhm.202001998 (PMC11468938; doi:10.1002/adhm.202001998)
Supplement: Supplementary file 1 — Supporting Information [file ADHM-10-2001998-s001.pdf]

© 2021 Wiley-VCH GmbH

ADVANCED HEALTHCARE MATERIALS

Supporting Information

for *Adv. Healthcare Mater.,* DOI: 10.1002/adhm.202001998

Hydrodynamic mixing tunes the stiffness of proteoglycan-mimicking physical hydrogels

James P. Warren, Danielle E. Miles, Nikil Kapur, Ruth K. Wilcox, and Paul A. Beales**

Hydrodynamic mixing tunes the stiffness of proteoglycan-mimicking physical hydrogels

James P. Warren, Danielle E. Miles, Nikil Kapur, Ruth K.Wilcox, Paul A. Beales**

Dr. J.P. Warren, Dr. D. E. Miles and Dr. P. A. Beales

School of Chemistry, University of Leeds, Leeds, LS2 9JT, UK.

Dr. J. P. Warren, Dr. D. E. Miles, Prof. N. Kapur and Prof. R.K. Wilcox

School of Mechanical Engineering, University of Leeds, Leeds, LS2 9JT, UK.

Dr. J. P. Warren, Dr. D. E. Miles and Prof. R.K. Wilcox

Institute of Medical and Biological Engineering, University of Leeds, Leeds LS2 9JT, UK.

Dr. P. A. Beales

Astbury Centre for Structural Biology, University of Leeds, Leeds, LS2 9JT, UK.

Dr. P. A. Beales

Bragg Centre for Materials Research, University of Leeds, Leeds, LS2 9JT, UK.

** Correspondence: R.K.Wilcox@leeds.ac.uk; P.A.Beales@leeds.ac.uk*

Supplementary Information

S1.1 Rheology : Example data set (P_{11}-12 : CS 1:20 Ratio, 30s)

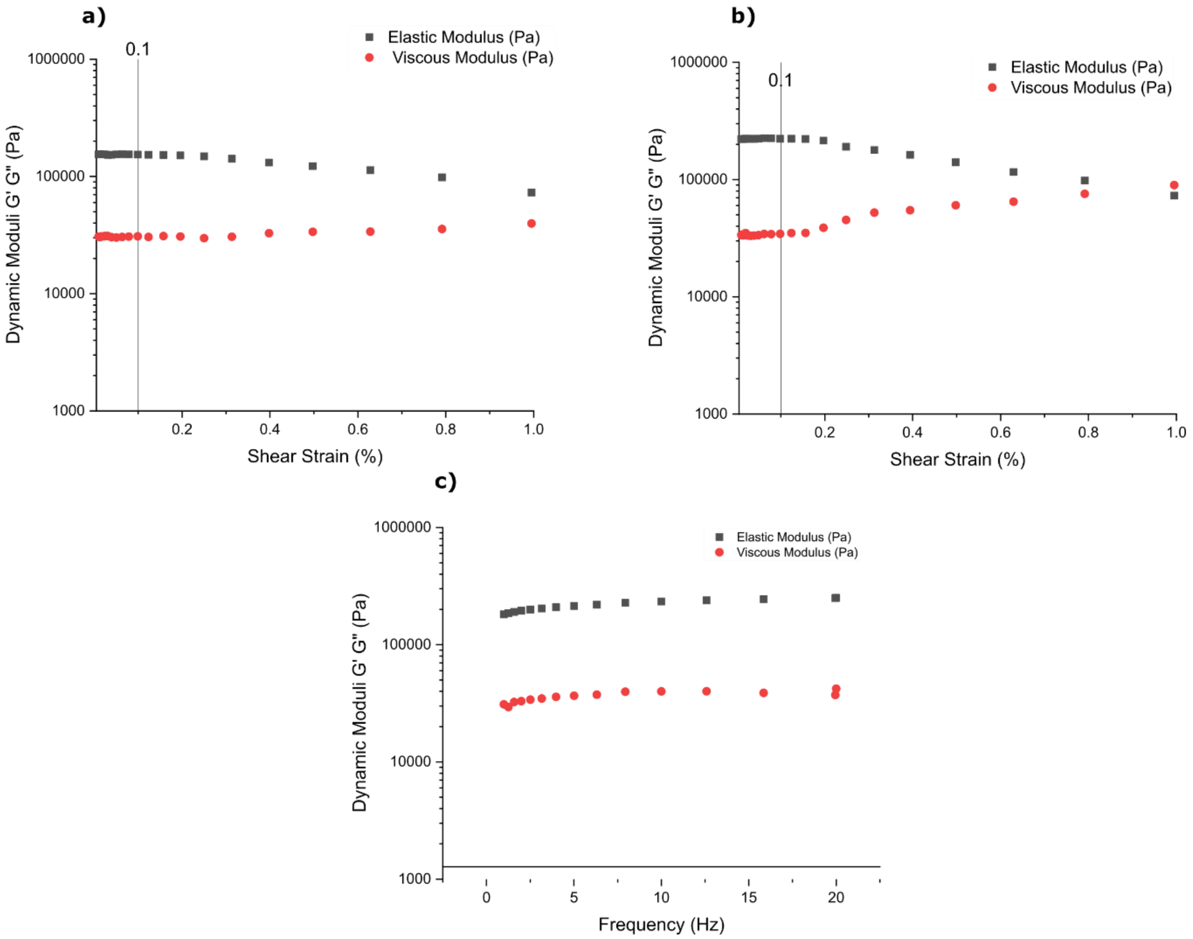

Figure S1: Representative rheological data illustrating process to measure dynamic moduli of hydrogel samples. a) Amplitude sweep at 1 Hz from 0.01-100% - sweep stopped at 1%, above which, the mechanical moduli become strain-dependent, indicating they are no longer in the linear viscoelastic regime. b) Amplitude sweep at 20 Hz from 0.01-100% - sweep stopped at 1%, above which, the mechanical moduli become strain-dependent, indicating they are no longer in the linear viscoelastic regime. c) Frequency Sweep from 1 Hz to 20 Hz (0.1% strain level)

S1.2 FTIR Spectra: Amide I band fitted

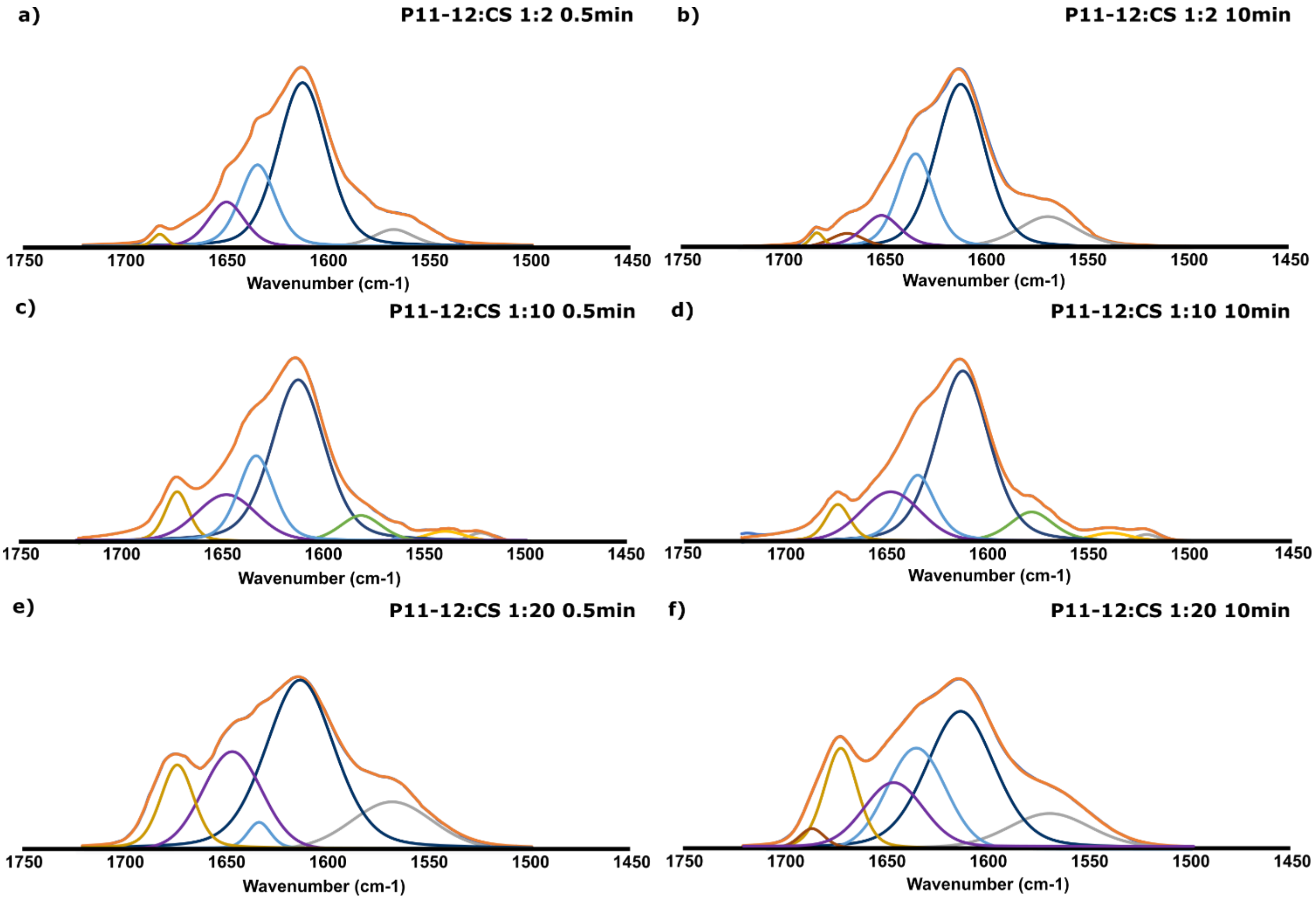

Figure S2: Amide I band fitted normalised spectra of hydrogels at different compositions and vortex durations to highlight shifts in fitted peaks from analysis. The two ends of the vortex duration range were chosen to illustrate the negligible change in the shape of the resulting spectral envelope and minor changes in the peaks fitted as part of processing. a) Ratio 1:2 peptide:CS 0.5min, b) Ratio 1:2 10min, c) Ratio 1:10 0.5min, d) Ratio 1:10 10min, e) Ratio 1:20 0.5min, f) Ratio 1:20 10min.

S3.1 High Resolution TEM images

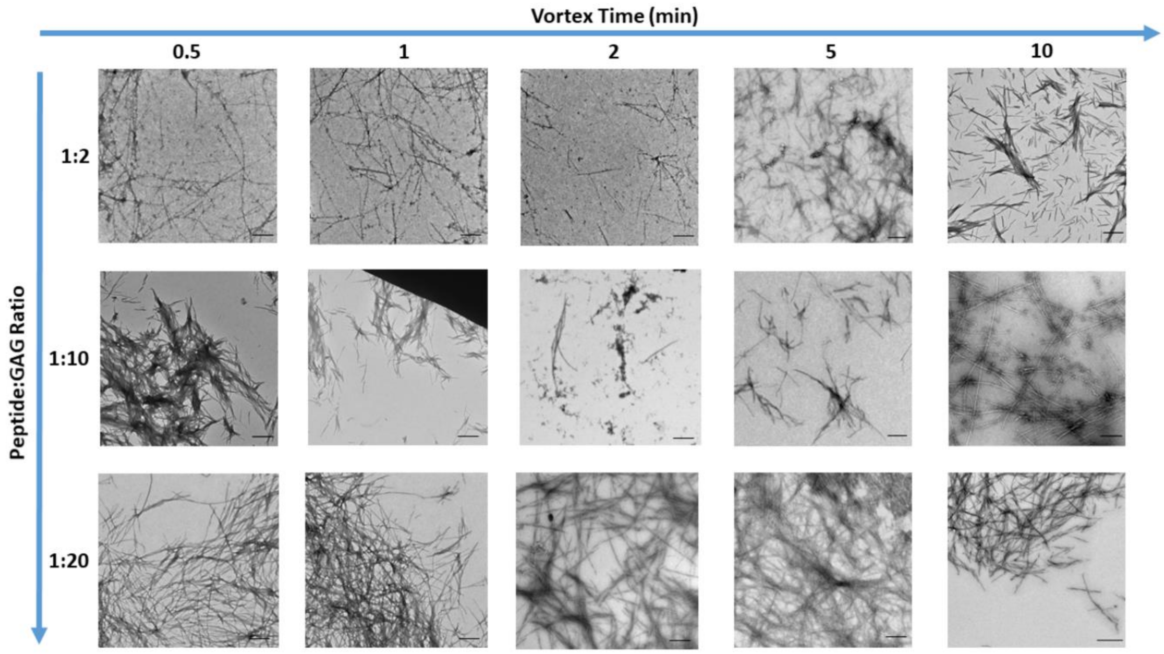

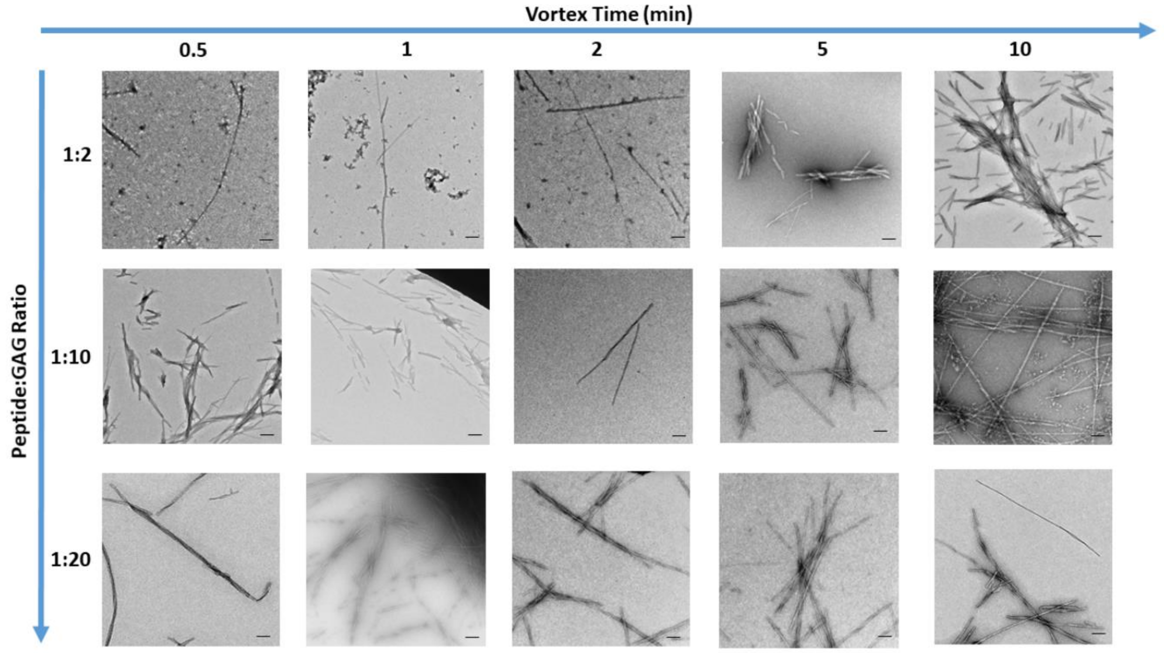

Figure S3:a) Micrographs of all vortex durations and compositions. Magnification: 4,000x, Scale bar: 500 nm. b) Higher magnification micrographs of hydrogels at various compositions and vortex durations. Magnification: 12,000x, Scale bar: 100 nm.
